# Video-Based Intervention for Improving Maternal Retention and Adherence to HIV Treatment: Patient Perspectives and Experiences

**DOI:** 10.3390/ijerph18041737

**Published:** 2021-02-10

**Authors:** Steven Masiano, Edwin Machine, Mtisunge Mphande, Christine Markham, Tapiwa Tembo, Mike Chitani, Angella Mkandawire, Alick Mazenga, Saeed Ahmed, Maria Kim

**Affiliations:** 1 Baylor College of Medicine Children’s Foundation Malawi, PBAG B397, Lilongwe, Malawi; edwinmasese@gmail.com (E.M.); mmphande@tingathe.org (M.M.) ttembo@tingathe.org (T.T.); mchitani@tingathe.org (M.C.); amkandawire@tingathe.org (A.M.); amazenga@tingathe.org (A.M.); sahmed@tingathe.org (S.A.); 2 Center for Health Promotion and Preventive Research, Department of Health Promotion and Behavioral Sciences, The University of Texas, Houston, TX 77030, USA; Christine.Markham@uth.tmc.edu; 3 Section of Retrovirology and Global Health, Baylor College of Medicine, Houston, TX 77030, USA

**Keywords:** VITAL Start, HIV, adherence, retention, Malawi, sub-Saharan Africa, acceptability

## Abstract

VITAL Start is a video-based intervention aimed to improve maternal retention in HIV care and adherence to antiretroviral therapy (ART) in Malawi. We explored the experiences of pregnant women living with HIV (PWLHIV) not yet on ART who received VITAL Start before ART initiation to assess the intervention’s acceptability, feasibility, fidelity of delivery, and perceived impact. Between February and September 2019, we conducted semi-structured interviews with a convenience sample of 34 PWLHIV within one month of receiving VITAL Start. The participants reported that VITAL Start was acceptable and feasible and had good fidelity of delivery. They also reported that the video had a positive impact on their lives, encouraging them to disclose their HIV status to their sexual partners who, in turn, supported them to adhere to ART. The participants suggested using a similar intervention to provide health-related education/counseling to people with long term conditions. Our findings suggest that video-based interventions may be an acceptable, feasible approach to optimizing ART retention and adherence amongst PWLHIV, and they can be delivered with high fidelity. Further exploration of the utility of low cost, scalable, video-based interventions to address health counseling gaps in sub-Saharan Africa is warranted.

## 1. Introduction

Despite global progress in reducing the number of new HIV infections, mother-to-child transmission of HIV remains a critical challenge. Globally, nearly 160,000 children (0–14 years) were infected with HIV in 2019, with over half (84,000) of these infections occurring in Malawi and other countries in sub-Saharan Africa (SSA), primarily through vertical transmission of HIV [1]. Currently, antiretroviral therapy (ART) to prevent vertical transmission of HIV and optimize maternal and infant health outcomes is widely available, but pregnant and lactating women living with HIV must continue to receive and adhere to ART to realize these benefits. Adherence to ART is necessary to achieve viral suppression and confer maximal health benefits, including reduced risk of vertical HIV transmission [2,3,4,5].

Unfortunately, suboptimal maternal retention and adherence to ART remain significant global challenges to halting the HIV epidemic and eliminating vertical HIV transmissions [6,7]. It is estimated that 1.06 million, or about 92% of pregnant women living with HIV (PWLHIV), received ART in 2018 in SSA [1,8], but only 79% and 75% were retained in care at 6 months and 12 months, respectively [7]. In comparison, in the general adult population, the 12-month retention is estimated to be 81% [9]. In Malawi, the first nation to implement lifelong ART for PWLHIV, close to 90% of PWLHIV received ART, but 12 months after ART start, only 74 % remained alive and in care [10]. This issue is not unique to Malawi, as evidence from other countries within SSA demonstrates shortfalls in adherence and retention in the setting of preventing vertical HIV transmissions [11,12,13,14,15,16,17]. 

Several barriers preventing optimal retention and ART adherence in Malawi and other countries in SSA have been identified in both quantitative and qualitative studies [17,18,19,20,21,22,23]. Reported barriers include fears to disclose HIV status to a partner, lack of adequate information and supportive education about ART and associated side effects, lack of empowerment to persuade a partner to go for testing, lack of understanding about the benefits of taking ART while still strong and healthy, lack of understanding about how resistance develops and why one needs to take ART when healthy, lack of understanding of what needs to be done when one has missed a dose of ART, stigma, and insufficient support from healthcare workers. These challenges were further exacerbated by the COVID-19 pandemic, which limited face-to-face interaction between clients and providers to prevent further spread of the disease. Therefore, identifying effective, low-cost, and scalable interventions that address these barriers and can improve maternal retention and adherence remains a priority for both researchers and policymakers. However, finding such interventions has been challenging. 

To overcome these barriers and find effective, low-cost, easy-to-scale, and simple-to-implement interventions to improve both short term and long term maternal retention and adherence, Baylor-Malawi created a video-based intervention to deliver ART information and education to prevent vertical HIV transmissions to PWLHIV before ART initiation. The video intervention—known as VITAL Start (Video-based intervention to Inspire Treatment Adherence for Life)—aims to improve maternal ART retention and adherence through behavior change by providing a VITAL Start at the critical teachable moment before committing to lifelong ART. The messages in the video are woven into an entertaining drama. Baylor-Malawi decided to create the video intervention because many of the barriers to maternal retention and ART adherence related to lack of information and empowerment of women to prevent vertical HIV transmissions, as well as to help standardize pre-ART counseling and overcome shortages of skilled healthcare workers that provide pre-ART counseling. The intervention can be delivered by lay health care workers and does not require as much healthcare worker time as the standard of care—pre-ART counseling delivered using flipcharts, as recommended by the Malawi Ministry of Health [24,25]. On average, it takes 37 min to deliver the intervention (27-min video and 10 min post-video Q&A session) compared to the standard of care, which takes about two hours. Participants watch the video alone or with a partner during which time the healthcare worker waits outside or performs other tasks. 

The VITAL Start intervention is currently being evaluated in an NIH-funded multi-center individual randomized controlled, outcome assessor-blinded clinical trial at three government health facilities in Malawi [25]. The trial’s primary aim is to evaluate the effects of VITAL Start on maternal ART adherence and retention in HIV care [25]. The study includes pregnant women who are HIV positive by two antibody rapid tests, age ≥18 years or 16–17 years if married or have a child, understand Chichewa (the local language in Malawi), willing to provide informed consent, and intend to remain in the health center catchment area for ≥6 months. Women already on ART, with significant pre-existing psychiatric comorbidity, or who participated in the VITAL Start pilot are excluded [26]. At all three study sites, HIV testing of pregnant mothers is provided at the antenatal care (ANC) clinic and the pre-ART counseling (VITAL Start or standard of care) is delivered soon after a HIV diagnosis at the same clinic. Participants randomized to the intervention arm—VITAL Start—view the video on a tablet with headphones in a private room at the clinic while the healthcare worker waits outside or delivers other services. After watching the video, participants have a brief follow up one-on-one post-video discussion with the counselor. Participants randomized to the control group receive the standard of care (pre-ART counseling delivered by a healthcare worker using the National Counselling Flip Chart) [24,25]. Further details about the trial are documented elsewhere [25].

While the effectiveness of VITAL Start will be evaluated at the end of the trial, the current study seeks to understand women’s experiences with VITAL Start in terms of its acceptability, feasibility, fidelity, and perceived impact. Understanding these experiences can help to elucidate how the intervention works and identify opportunities to improve the intervention. Importantly, this study helps to explore the applicability of video-based interventions in resource-limited settings where the use of such interventions has been explored extensively. Exploring alternative ways of delivering health services in general, and HIV services in particular, carries additional significance in this era of the COVID-19 pandemic when minimal contact between clients and their providers is recommended to help minimize the spread of the disease.

## 2. Materials and Methods

### 2.1. Setting 

This qualitative process evaluation was conducted with women at all three sites of the VITAL Start study [25]. Two of the sites, Area 25 and Kawale, are large urban health centers in Malawi’s capital city, Lilongwe. The third site, Mangochi, is a busy district hospital in Southeastern Malawi. At the start of the VITAL Start study in 2018, ANC HIV prevalence at these clinics ranged from 11–13%, with approximately 60% (900/1500) of HIV-positive pregnant women not yet on ART.

### 2.2. Study Participants and Procedures 

Between February and September 2019, we conducted semi-structured interviews with a convenience sample of women enrolled in the trial who had received the VITAL Start intervention. Initially, women who had received VITAL Start were invited by phone to come to the facilities to do the interviews, 14 to 30 days after watching the video. A shorter recall period (14–30 days) was chosen because it was proximal enough to the receipt of the intervention yet provided participants with enough time to process the intervention and see how it might affect their lives [27]. However, once we realized that many women did not have phones we also started to invite women to participate at enrollment or month 1 follow-up. We continued to conduct interviews until we felt no new themes were emerging (saturation). We emphasize that all interviews were conducted in-person, but the invitations for the interviews were done either by phone or in-person. All interviewees were reimbursed for transport expenses.

To generate a richer discussion on topics of interest and elicit candid responses, open-ended questions followed by probing questions were used for the interviews. We developed an interview guide to ensure that the same general areas of information were collected from each participant. The interview guide was collaboratively developed by Malawian and US investigators, piloted among potential study participants, and further refined. Participants were asked about their views on the acceptability, feasibility, and fidelity of the video intervention, their perceptions about its strengths, motivational aspects, challenges, and benefits they experienced, and suggestions for improvement. Further, the interview items sought to understand if the video accurately communicated the intended educational messages and if there were any unintended messages conveyed. Participants were encouraged to frankly share positive and negative experiences. 

All interviews were conducted by Malawian research staff trained in qualitative methods who had prior experience with qualitative interviewing and human subjects’ research. Written informed consent was obtained from all participants before the interviews. The interviews—which lasted approximately 35 min—were conducted in Chichewa and occurred in a private setting within the health facility. In total, 132 out of 135 women who received the VITAL Start intervention were approached for the interviews. Of these, 37 (28%) returned for the interviews. Out of the 37 who returned, a total of 34 interviews were conducted: Area 25 (12), Kawale (11), and Mangochi (11). The study staff felt that three participants were still experiencing very intense emotions and would be unable to process the interview questions. Of the 34 women who returned for the interviews, only 3 had watched the video with their partners.

### 2.3. Ethical Approval

This study received ethical approval from the National Health Sciences Research Committee of Malawi (# 1593) as well as the Baylor College of Medicine’s Institutional Review Board (# H-39785).

### 2.4. Analysis

A qualitative thematic analysis was conducted utilizing Atlas.ti. The interviews, which were tape-recorded, were transcribed verbatim in Chichewa and translated into English. The thematic analysis of the data drew from predetermined and emergent themes and used *priori* core topical codes [28], guided by the constructs of interest: Acceptability, feasibility, fidelity, and perceived impact of the intervention. Ongoing comparison of codes helped refine subsequent interviews, allowing for emergent themes [29,30]. The research team followed a rigorous protocol to ensure the consistency and validity of coding during the qualitative analysis, although a formal inter-rater reliability statistic was not computed. We used an iterative process of discussions with co-authors and research team members to develop initial codes that were then reconciled into an agreed coding framework and subsequently applied to the data in the final analysis [31,32,33]. This approach has also been used in multiple studies of quality improvement in sub-Saharan Africa [32,33]. Coding was primarily conducted by two researchers, including one Malawian researcher, and the Malawi based research team (the principal investigator and site research staff) offered feedback via participation in debriefing sessions. 

For the purposes of this study, we used the following definitions of implementation outcomes adapted from Proctor et al. [34], Table 1. 

## 3. Results

The socio-demographic characteristics of the 34 women who received the VITAL Start and returned for the interviews are described in Table 2. The median age was 27 years (interquartile range: 22–32 years), the majority were married, housewives, with low-income levels, and were not the primary decision-makers at home. Nearly half (47%) had only primary or no education.

### 3.1. Women’s Perspectives on VITAL Start

Several findings emerged from the analyses, showing which aspects of VITAL Start were liked and valued as well as areas where further improvements might be suitable. The emergent themes within each implementation outcome (acceptability, feasibility, fidelity, and perceived benefit) and areas for improvement are presented below with supporting excerpts from the interviews.

#### 3.1.1. Acceptability

“…the video was good…”

Acceptability was examined by asking the women whether they liked the video or had any questions and concerns about the video. Overall, participants felt VITAL Start was acceptable. Participants liked that the intervention helped reduce their anxieties and fears related to a HIV diagnosis, met their expectations, dispelled some myths, and gave them hope. They also liked the post-video session, which helped to reinforce the message and the video setting, which they could easily relate to.

*VITAL Start reduced anxiety and fears after diagnosis:* When asked what fears or questions they had upon diagnosis, participants said they had concerns that ranged from the social stigma associated with being HIV-infected, end-of-life ideation, fear of being different from other people, and fear of getting ill frequently. Some participants reported being told that ART changed one’s appearance, while others felt unprepared to take ART daily for life. Participants reported that VITAL Start helped to address these fears and concerns.

*“…the video was good, (‘ndinaiwona bwino’, which renders it as good) there were stories of how to take medication …it helped to get rid of them all [the fears] …”*

[Participant 3026]

*“…I had fear because we were together with my husband (watching video); therefore, I thought that maybe we would have problems…maybe the marriage would end. ...; however, when we went home, it is when he reminded me that it’s time to receive/collect medication… Since then, I have been very happy…”*

[Participant 2087]

*VITAL Start met participant expectations and gave them hope.* In addition to reducing fears, participants also reported that VITAL Start met their expectations, helped them to understand their situation better, and gave them hope that they could stay healthy and live normal lives despite being diagnosed with HIV. The video also dispelled myths about the health of people living with HIV.

*“…yes, it [the video session] met my expectations… it managed to help me to understand my status and also how I am supposed to live…”*

[Participant 2087]

*“…when I watched it, I was encouraged that I do not have any problems…”*

[Participant 1086]

*“…when I watched the video, it showed me that I am not very different from the people that are normal [those without HIV] … someone does not get sick frequently, therefore I thought that the only difference would be taking medication daily. Therefore, I saw that I am a normal person…”*

[Participant 2087]

*Post-video discussion reinforced the VITAL Start message:* Some participants reported that the post-video sessions, which involved a sit-in with the counselor, complemented the video session well. Participants liked the post-video discussion because it echoed what they heard in the video and presented an opportunity to clarify any lingering issues.

*“…because, at first, they asked questions on a thing that I had not learned yet, and when I watched the video, it was like they (the counselor post-video) were repeating so that I should remember what I had watched…”*

[Participant 2087]

*A more believable story because of the use of relatable video settings*: Most participants found the video setting relatable (use of common places like the market and people doing everyday activities)—which made the messages more believable, appropriate, and well-received,

*“…the video was shot well because at the market is where men work, they fix bicycles and buy things, the other place is at home, and the other place is the hospital where people receive counseling. The video showed all the places…”*

[Participant 2087]

#### 3.1.2. Feasibility

“…everything went well…”

Feasibility was examined by asking questions about the suitability, practicability, and utility of the video. The women reported that VITAL Start was feasible given that they had a positive experience overall. Specifically, participants said that video instructions were clear, they did not encounter any challenges when using the equipment, and using the equipment was comfortable. They also valued the privacy of watching the video alone in a room given that clinics are crowded places and lack privacy. They appreciated that this arrangement prevented inadvertent disclosure of their HIV status.

*“…Everything seemed well. They told me what to do if it pauses, but it did not. I watched it very well up until I finished…”*

[Participant 1057]

*“…it was good because there was no other person who disturbed me when I was watching the video. I watched it freely…”*

[Participant 1093]

*“…I felt very sweet [an exaggerated expression of feeling good]…because if I was to watch it with someone else, my friend would have gone out and spread the bad news…so I was very happy because the things I watched, I was watching alone…”*

[Participant 2047]

#### 3.1.3. Fidelity of Delivery

“…the most important message was adhering to medication…”

Fidelity of delivery was assessed by invoking retrospectively whether key messages or concepts in VITAL Start were delivered as intended. The majority of the women were able to recall key messages from the video; three women (9%) were unable to recall all the key messages. Specifically, the women felt the video increased their ART related knowledge, improved their self-efficacy, and encouraged partner disclosure. 

*Increasing ART knowledge:* Participants reported positive messaging about ART in general, and also on the key counseling messages regarding HIV diagnosis and treatment. This included the benefits of ART for preventing vertical HIV transmissions and maternal health, the importance of adherence to prevent drug resistance, possible side effects, strategies to adhere to ART, and partner disclosure.

*“…they (in the video) were talking about the medication so that one should be well in her life …and life should go on well and the expected child as well. And if we stop taking medication, then the virus will wake up and get to the child, and the child will not be alright and later the child is born infected…”*

[Participant 3048]

*“…the most important message (from watching the video) was adhering to medication so that I can protect the child that I am expecting…”*

[Participant 3108]

*Improving self-efficacy or changing behavior:* Participants reported the intervention helped establish adherence promoting behavior such as setting aside a specific time for taking the medicines, with the majority choosing to take the medicines around dinner time. Other participants reported selecting a safe and easy-to-remember location in the house to keep their medication.

*“…the ones I still remember are: I am supposed to go to the hospital to collect drugs in good time, on a scheduled day/the day you were told. One is not supposed to miss a dose, one is supposed to adhere to medication…”*

[Participant 2055]

*“…oh, this helped us in a lot of things: 1, about diet. 2, about taking medication. 3, taking care of oneself. Yah, it taught us a lot…”*

[Participant 2141]

*Encouraging partner disclosure:* Participants also reported feeling that VITAL Start encouraged partner disclosure. Most participants reported that the video helped them to find a starting point for disclosing, as well as on how to say what they wanted to say.

*“…it (the video) encouraged me that I should not be afraid. At first, I was afraid that what am I going to do, and what do I do? But after watching the video to say, no you should not worry and I did not get worried; when I reached home, I started explaining (to my husband)… ”*

[Participant 1081]

*“…it (the video) helped me to have the courage that I should open up to my partner and also to a friend …”*

[Participant 2055]

*“So after watching the video, I saw that they have taught me ways of making him understand and how to approach my husband…”*

[Participant 1051]

*Recall challenges:* Three participants had challenges recalling all the key messages from the video. For example, when asked what the main message was in the video, two participants said they could not recall the key messages. These participants, however, were still able to recall other messages. 

#### 3.1.4. Benefits or Perceived Impact of VITAL Start

“…Ah, let us plan a day I should also go for HIV testing and counseling”

The perceived impact of VITAL Start was assessed by understanding participants’ actions or behavioral responses after watching the video. Following the intervention, several actionable responses or behavioral changes emerged, including more women disclosing their HIV status to their partners, greater partner engagement, and more women feeling empowered. However, a handful of women still feared to disclose to their partners. 

*Cue to action for partner disclosure:* When asked to describe any actionable responses or behavioral change participants experienced as a result of the video intervention, actionable areas were disclosing their HIV status, partners going for HIV testing and counseling, and partner support in medication adherence. Some participants reported that they were pleasantly surprised by the overwhelmingly positive response and support they received from their partners after disclosing and asking their partners’ to get tested as well.

*“… so when I reached home after watching that video…I started asking him questions that “are you ready to go for testing?” He responded that “Yes I am ready”. “What if we are found positive, what will be your thoughts?” Then he kept quiet; he did not answer me. So the issue was continued in the evening that “What are your thoughts on that issue we were discussing?” He responded that “Whatever the case will be, I will accept it because I am not the first one…”*

[Participant 1081]

*“…eh, because my husband was refusing to get tested, so when I left here, I told him. So he said that “Ah, let us plan a day I should also go HIV testing and counseling.”*

[Participant 2122]

*“… He [my husband] asked me why I was taking time at the hospital, I explained. When I got home, he welcomed me happily. I told him I was not going to take the medicine. He asked if I had been counseled. I said I would take the medicine the following day. He responded that I shouldn’t do that but wake up. He woke up and handed me Super Shake. When I take medicine with water I vomit. So he opened the bottle and handed it to me and then what?... I drank it. Yesterday, he mentioned that it is unfortunate that today is Sunday, he would have loved to know his HIV status. Also, he encourages me every evening to take my medication so that we protect our unborn baby. This makes me happy…”*

[Participant 2142]

*“… When I was coming this morning, he was the one who readied me…”*

[Participant 2142]

*Lingering fears to disclose:* While the video helped to alleviate the women’s fears around HIV status disclosure and made them feel prepared to disclose, four women (12%) could not disclose their HIV status to their partners. They were worried or unsure about how their partners would react if they disclosed their status. Despite these fears, some of the participants hoped that someday they would have the courage to disclose or wait until the day their partner is in a good mood.

*“…it is about the message; the person in the video told her friend while I have not yet told any friend, or even a relative. I will tell them in the future and I will tell my relatives how things are…I had intentions or thoughts [to disclose] because he is my husband; he is the one I would tell first. However, during that time, I was thinking about how I could make him understand…”*

[Participant 1051]

*“…it [watching the video] removed my fears, but I just didn’t know how to start [to disclose]. And when I see him, something bothers me, but what challenges me is how I am going to explain all these things…I believe that God will strengthen me so that one day I shall tell him…”*

[Participant 1057]

*“…I was scared because, for one to tell the partner [that they are HIV positive], that means you are close to him. Maybe if he told me that he will marry me after making me pregnant, then you can tell him…But if we were married, I would have told him to come with me and get tested…”*

[Participant 3025]

*“…To tell him… I will know a day how he will carry (conduct) himself, that day I will explain to him the truth, “I went to the clinic, they found me positive … this is the (clinic) book. But because of the way you behave (react), I was afraid to explain to you, but today this is it”…”*

[Participant 2123]

*Greater partner engagement:* Participants who watched the video with their partners reported that it was very beneficial for their partners to view the video and that the video enhanced partner engagement.

*“…there were similarities (the video and my circumstances) when the man in the video refused to go to the hospital… my husband was telling me to wait, that he should find time to excuse himself at work, and when he did, we went [together]. While in the video, the husband refused to go… that video story relates to me….my husband is someone who has changed the way he talks from that day up until now; he is very loving than how he used to be in the past. It seems like the video taught him a lot of things… the way we stayed at home at first, we never chatted together; however, I now have enough time to chat with him when he calls me and sit and chat together. And I can see that things have changed in my life …until now…”*

[Participant 2087]

*Increasing empowerment:* Some participants described how the video empowered them to take measures to ensure treatment adherence, including using times for news bulletins as a reminder.

*“…My procedure (routine put in place to help with adherence), I cannot forget to take the drugs because I am HIV positive, and I have drugs in the house…I cannot forget no matter how busy I may be. I can stop what I am doing. Taking drugs cannot take more than 30 min, it is not possible…”*

[Participant 1081]

*“…I like listening to the radio, so the time I have set for taking medication is 7 o’clock. When the time is 7 o’clock, I am supposed to go and take medication before I start listening to the news…”*

[Participant 1057]

#### 3.1.5. Opportunities for Improvement and How a Similar Video Intervention might be Used

“…But if you could reach out to the people in these villages, we could all be liberated”

Finally, the participants suggested aspects of the video that might be improved, modified, or expanded. They also suggested diseases and medical conditions, other than HIV/AIDS, for which a similar type of video might be used. These suggestions included using the video in communities, creating an audio version of the counseling that can be aired on radios, and developing a similar video for people with cancer or tuberculosis. The opportunity for improvement was examined by asking questions about whether there should be any modifications that would make the intervention more palatable, attractive, or valuable, and how a similar type of video intervention might be utilized for the benefit of other types/categories of patients and settings. 

*Expanding to communities and using radio messaging:* Some participants suggested that the video intervention should be expanded to communities, particularly among folks who do not make it to HIV testing sites. Reaching out to possible beneficiaries may involve the use of other media such as radio messaging:

*“Maybe the sick people who are in the villages who do not [want to] go to the hospital, those who do not want to know their status, those who may be visited and shown the video, perhaps they may be encouraged to get their blood tested…”*

[Participant 1057]

*“…if you could reach out to these patients in the villages. There are people in these villages who are afraid. But if you could reach out to the people in these villages, we could all be liberated…It is not everyone who went to school; some of us didn’t. When people hear the word hospital, it is a very frightening word. Transporting oneself there [the hospital] is a problem…A lot of people cannot manage to walk to get there; if you can be reaching out to them and counsel them, they could be happy people and very content.”*

[Participant 2142]

*“…you should even put it [the counseling] on the radio so that other people can also listen. Considering the people who have not tested for HIV, if they could listen to this message or if they could watch the video, it means it could change people’s lives…”*

[Participant 1093]

*“… It is not only HIV positive people who should watch this video, no. Any person who is HIV positive or negative…”*

[Participant 1093]

*Creating similar videos for people with long-term conditions:* When probed on which other individuals are likely to benefit from a video intervention, some participants suggested patients with other long-term conditions, such as cancer:

*“…maybe cancer [patients], [people with] epilepsy; people can have video counseling since it shows everything very well…”*

[Participant 3026]

*“…such as those with TB (tuberculosis) and malaria…”*

[Participant 3025]

## 4. Discussion

Identifying effective, low-cost, and scalable interventions that can improve maternal retention and adherence, particularly in sub-Saharan Africa where the majority of people with HIV live, remains a priority in a global quest to achieve a HIV-free generation. However, finding such interventions has been challenging. This study explored the perceptions and experiences of PWLHIV who received VITAL Start—a video-based intervention to deliver ART information and education to prevent vertical HIV transmission to PWLHIV before ART initiation. Specifically, we sought to understand the intervention’s acceptability, feasibility, fidelity, perceived impact, and aspects of the intervention that might need to be improved. Participants (all women) found the intervention highly acceptable—it helped to allay their fears and concerns about their HIV diagnosis and depicted the “way of life” for most of them. The women also reported that watching the video went well without any glitches, which suggests that the video was feasible. We also found that the fidelity of delivery was good, given that the women were able to recollect key messages and concepts from the video and reported that the video helped them to adhere to ART. Additionally, participants reported that the video had a positive impact on their lives as it gave them the courage to disclose their HIV status to their sexual partners who, in turn, encouraged them to adhere to ART and showed a willingness to undergo HIV testing and counseling. At the same time, the findings highlight opportunities to further optimize VITAL Start and expand the use of similar interventions in this setting. These opportunities include creating other videos to support or provide education to people with long term conditions and implementing the interventions in the communities, not only in clinics.

Overall, these findings suggest that VITAL Start met its intended purpose and highlight the importance of creating video interventions including settings and characters with whom the targeted audience can identify. The women were satisfied with the video content; they could relate to the setting of the video as it was shot locally with characters plying typical trades and living a type of life that many people in Malawi and similar settings live. Thus, the women could map the video content to their circumstances by comparing and contrasting the characters represented. The post-video session with a counselor and the post-video script ensured that the key messages from the video were not lost. The video’s intended message included empowering women to be able to freely talk about living with HIV, teaching them the benefits of ART adherence, and how a reminder system could help ensure adherence. Some of the reminder systems reported included identifying common familial routines convenient for them to be taking ART, for example, mealtimes, a favorite program on television or radio, or just before bedtime. An additional take-home message was about encouraging the participants to disclose their HIV status to their loved ones who, in turn, would support them as needed. Indeed, what the women reported suggests that those who disclosed received support from the partners, including being encouraged to adhere to ART. Finally, the video delivery was convenient, with the participants saying the tablet was easy to use. Furthermore, they appreciated the privacy of individualized viewing and the use of headphones, which eliminated inadvertent disclosure of HIV status and the stigma associated with watching the video. 

While the fidelity of delivery of the intervention was good, we note that three women (9%) had difficulties recalling specific messages from the video, although this is not inconsistent with the literature [35,36,37,38,39]. In one of the earliest studies on the maintenance of knowledge in Spain, up to 88% of college men and women recalled new material after 30 days [39]. The three participants still remembered a substantial amount of information, and there was no pattern to what they could not recall. This was not unexpected, for two reasons. First, most of the participants were newly diagnosed with HIV and, inevitably, must have been shocked by the news, and their ability to process and assimilate information in the video regardless of the recall period might have been compromised [40,41]. Second, nearly half of the participants had little or no education, and the evidence suggests that education helps with recall [27,42]. Nonetheless, it is encouraging that 91% of the women recalled all the key messages. This suggests that the storylines of the 27-min video and the 10-min post-video Q & A session were sufficiently engaging to make 91% of the participants remember the key messages. Moreover, these recall rates are as good as those reported in one-on-one or group counseling sessions in other healthcare services, including recall of the maternal information provided during prenatal and postnatal care [35,36,38]. 

One of the aims of VITAL Start was to help PWLHIV disclose their status to their partners, and our findings suggest that nearly 90% disclosed—which compares favorably with reports from many studies in sub-Saharan Africa aimed to improve HIV status disclosure among sexual partners [43,44], as well as other video-based interventions globally [45]. For example, cognitive-behavioral support groups reported disclosure rates of 81–87% in South Africa [46,47] and slightly over 90% in Zimbabwe [48]. The study in Zimbabwe likely reported higher disclosure rates because the support groups used various learning techniques such as discussions, behavior modeling, songs, and role-playing to reinforce the message, empower participants, and equip them with negotiation skills. In the current study, reasons for nondisclosure ranged from not having stable relationships to fearing reactions and violence from partners. These reasons have also been reported in the literature over the past two decades [44,49,50,51,52]. Our findings are encouraging as they suggest that VITAL Start made the women feel empowered and encouraged them to disclose. Therefore, VITAL Start is potentially an additional tool or medium that can be used to help with disclosure, knowing that it can achieve similar or higher disclosure rates. 

We also highlight one unexpected positive finding—watching the video with a partner was rewarding as it increased partner engagement. This was somewhat unexpected because we have observed that men in Malawi do not often accompany their spouses to the clinics, and if they do, they (men) appear to pretend to be in a hurry. Although the video was designed to provide education on the prevention of vertical HIV transmission to PWLHIV and empower them to disclose their HIV status to partners, the finding suggests that interventions targeting women should keep both women and their partners in mind to optimize the intervention’s benefits.

Overall, these findings reinforce emerging literature promoting the use of video interventions in healthcare. Video interventions have been used successfully in sexually transmitted disease clinics [53,54,55,56], palliative care clinics [57], for home visits by community health workers [58], and in both prenatal and HIV posttest counseling [59,60]. As in these studies, our study suggests that the VITAL Start intervention is acceptable, feasible, and impactful, as the majority of the women reported positive behaviors. Compared to other video-based interventions, the short post-video Q & A session likely helped to obtain the positive results we have reported. During the Q & A sessions, a healthcare worker and the client discussed the steps the client would take after watching the video in a way that engaged the women and empowered them to decide and pursue their desired outcome. Furthermore, the post-video session assured the women of provider support every time they came to the facility for services. 

It is worth noting that the current study, although largely exploratory, reports on four implementation outcomes (acceptability, feasibility, fidelity, and impact), while the majority of studies that examine intervention implementation in healthcare, including the use of evidence-based interventions in HIV care, only report on a few outcomes; most only report acceptability [61]. By reporting on these four implementation outcomes, we give a more complete understanding of the implementation of VITAL Start, which is useful when making scale-up considerations. 

This study has several limitations. First, only about a quarter of eligible participants—those who received the VITAL Start intervention—returned for interviews, suggesting that our study might have suffered from selection bias. To better understand the nonresponses, we compared the characteristics of women who returned for the interviews and those who did not return to identify any selection bias issues. We found that the demographic characteristics of responders did not differ significantly from those who received the standard of care. Further, we compared the incidence of intimate partner violence—one of the post-intervention outcomes at month 1—among those who returned for the interviews and those who did not. We found that the incidence of intimate partner violence across three categories (emotional, physical, and sexual abuse) did not differ significantly among participants who returned for the interviews and those who did not. This suggests that those who failed to return for the interviews did not do so because of negative or adverse outcomes post-intervention, for example, when disclosing status to a partner. Therefore, despite some women not returning for the interviews, there is no evidence of selection bias; our findings are thus still representative of not only the women who received the VITAL Start intervention but also the entire study cohort.

The second limitation is that we were unable to stratify the analyses by the experiences of participants who watched the video with their partners and those who watched alone. This is because the number that watched the video with their partners was very small (3/34). Notwithstanding, participants who watched the video with their partners reported having a good experience and receiving partner support in ensuring that they adhered to ART. But as more data from the VITAL Start trial become available, this will be one area of further inquiry. Beyond what the participants reported during the interviews, we will look at whether verifiable outcomes such as ART adherence (measured by the concentration of tenofovir-diphosphate) and viral suppression among participants who watched the video with their partners differ from those of participants who watched the video alone.

The third limitation is that our findings may not be generalizable to all PWLHIV in Malawi—a limitation inherent in qualitative research designs [53,62,63]. The VITAL Start study and, hence, the interviews were conducted in three sites only, and our sample was largely composed of married women or those with partners. We acknowledge this limitation, but we argue that the three sites—a health center, a community hospital, and a district hospital in two of the most populous regions and with the highest proportions of people living with HIV [64]—reflect the healthcare system in Malawi and the country’s HIV profile. Besides, the number of interviews conducted was similar across the three sites and the responses had similar emergent themes regardless of site or marital status. This suggests that our findings may still be generalizable to the majority of PWLHIV in Malawi and perhaps other resource-limited settings. 

A major implication of our findings is that the video intervention can help to improve overall service delivery, and this can be achieved in two ways. First, the video saves time; it requires much less time for direct interaction between the client and provider compared to the standard of care [26]. The saved time allows the provider to focus on service delivery areas that otherwise would not have been attended to or would have been attended to without due diligence. Should a VITAL Start evaluation find it to be at least as effective as the standard of care, the benefits or value of the time saved in health service delivery can be substantial in Malawi and similar settings where human resource shortages relating to health care are acute. Moreover, some women suggested that using the video in communities—not just clinics—might benefit patients with similar conditions who fail to come to the clinics for testing and treatment. Although the women did not propose the settings or conditions under which this would be accomplished, the fact they brought it up suggests that video interventions are likely to remain acceptable to the majority of Malawians. This is encouraging because the continued or accelerated use of video interventions may, in the long term, help to alleviate shortages of human resources for health. Second, the video intervention could improve overall service delivery because it standardizes and consolidates key messages and delivers these messages consistently, so that the women found VITAL Start acceptable in content and delivery. The lack of standardization in traditional counseling suggests that the margin for error and inconsistent messaging is high, resulting in some women not receiving the right information on ART adherence and prevention of vertical HIV transmissions, which unfortunately may derail efforts to achieve a HIV-free generation. 

## 5. Conclusions

To eliminate mother-to-child transmission and, therefore, achieve a HIV-free generation, it is critical to find low-cost, easy-to-scale, and acceptable interventions that can increase ART adherence among PWLHIV and improve retention in HIV care. This qualitative study shows that a short video on ART and education to prevent vertical HIV transmission followed by a quick Q & A session is an acceptable, feasible, and impactful intervention to use among PWLHIV. The video intervention encourages HIV status disclosure to sexual partners and results in behavioral changes that impact ART adherence because it addresses the key issues in the ART cascade—from initial HIV diagnosis to achieving viral suppression. While considerations to scale up the intervention may have to wait until the effectiveness of VITAL Start compared to the standard of care is reported, our findings on the four implementation outcomes (acceptability, feasibility, fidelity, and impact) of VITAL Start are nonetheless encouraging. The findings suggest that solutions to the problems facing the optimal delivery of ART and prevention of vertical HIV transmission may be within reach. This is particularly true for many countries in sub-Saharan Africa, where most people with HIV live; mother-to-child transmission of HIV is still high, and human resources shortages continue to hinder the delivery of healthcare services. Furthermore, as suggested by the participants, future research could focus on exploring the feasibility of expanding the video intervention to communities to benefit people with similar conditions who fail to come to the clinics for testing and treatment.

## Figures and Tables

**Table 1:** Definitions of implementation outcomes.

Implementation Outcome	Definition
Acceptability	Participants’ perception of an intervention as agreeable, palatable, or satisfactory. Operationalized by assessing participants’ knowledge of, or direct experience or satisfaction with, various dimensions of the VITAL (Video-based intervention to Inspire Treatment Adherence for Life) Start intervention: the video content, complexity, and/or comfort. Examined by how participants experienced these elements, using codes such as comfortable/uncomfortable/troubled/satisfied/happy/got answers to their questions, concerns, or fears.
Feasibility	The extent to which a new treatment or an innovation can be successfully used in terms of ease and convenience, or carried out within a given setting. Operationalized through participants’ perceptions regarding the suitability, practicability, and utility of the video intervention in the manner it was made and delivered.
Fidelity	The degree to which video messages were received as intended by the program developers. Assessed by invoking retrospectively whether key messages or concepts in VITAL Start were delivered as intended.
Benefits and perceived impact	The actionable response(s) the participants had as a result of the intervention. Assessed by understanding participants’ actions or behavioral responses after watching the video.
Opportunities for improvement	Participants’ actionable suggestions to modify or improve the intervention. Investigated how the video might be improved by coding around whether there should be any modifications that would make the intervention more palatable, attractive, or valuable. Sought to understand patient perspectives on how a similar type of video intervention might be utilized for the benefit of other types/ categories of patients and settings, or further scaled up.

**Table 2:** Demographic characteristics of recipients of VITAL Start and participating in in-depth interviews (*n* = 34).

Characteristic	Frequency	Percentage *
Age	28	
Mean (SD)	27	(6.3)
		
Marital status		
Married/boyfriend	30	88.2
No partner	4	11.8
		
Level of education		
None	3	8.8
Primary	13	38.2
Secondary	15	44.1
Postsecondary	3	8.8
		
Income levels (in MK)		
<49,999	20	58.8
50,000–99,999	9	26.5
100,000–249,999	4	11.8
>250,000	1	2.9
		
Occupation		
Business (self-employed)	6	17.7
Employed (formal sector)	2	5.9
Farming	2	5.9
Housewife	24	70.6
		
Decision-maker?		
Yes	6	17.7
No	28	82.4

* The percentages may not add up to 100 due to rounding.

## Data Availability

The data presented in this study are available on request from the corresponding author. The data are not publicly available due to ethical reasons.

## References

[1] UNAIDS (2019). Fact Sheet—World AIDS Day 2019. https://www.unaids.org/sites/default/files/media_asset/UNAIDS_FactSheet_en.pdf.

[2] Ambrosioni J., Calmy A., Hirschel B. (2011). HIV treatment for prevention. J. Int. AIDS Soc..

[3] Cohen M.S., Gay C.L. (2010). Treatment to prevent transmission of HIV-1. Clin. Infect. Dis..

[4] Granich R., Crowley S.P., Vitoria M., Lo Y.-R., Souteyrand Y.P., Dye C., Gilks C., Guerma T., De Cock K.M., Williams B.G. (2010). Highly active antiretroviral treatment for the prevention of HIV transmission. J. Int. AIDS Soc..

[5] Montaner J.S., Hogg R., Wood E., Kerr T., Tyndall M., Levy A.R., Harrigan P.R. (2006). The case for expanding access to highly active antiretroviral therapy to curb the growth of the HIV epidemic. Lancet.

[6] Vrazo A.C., Firth J., Amzel A., Sedillo R., Ryan P.B., Phelps B.R. (2018). Interventions to significantly improve service uptake and retention of HIV-positive pregnant women and HIV-exposed infants along the prevention of mother-to-child transmission continuum of care: Systematic review. Trop. Med. Int. Health.

[7] Knettel B.A., Cichowitz C., Ngocho J.S., Knippler E.T., Chumba L.N., Mmbaga B.T., Watt M.H. (2018). Retention in HIV Care During Pregnancy and the Postpartum Period in the Option B+ Era: Systematic Review and Meta-Analysis of Studies in Africa. J. Acquir. Immune Defic. Syndr..

[8] WHO (2019). Prevention of Mother-To-Child Transmission (PMTCT). https://www.who.int/gho/hiv/epidemic_response/PMTCT_text/en/.

[9] Fox M.P. (2015). Retention of adult patients on antiretroviral therapy in low-and middle-income countries: Systematic review and meta-analysis 2008–2013. J. Acquir. Immune Defic. Syndr. (1999).

[10] Ministry of Health (Malawi Government) (2018). Malawi Integrated HIV Program Report July–September 2018.

[11] Napúa M., Pfeiffer J.T., Chale F., Hoek R., Manuel J., Michel C., Cowan J.G., Cowan J.F., Gimbel S., Sherr K. (2016). Option B+ in Mozambique: Formative Research Findings for the Design of a Facility-Level Clustered Randomized Controlled Trial to Improve ART Retention in Antenatal Care. J. Acquir. Immune Defic. Syndr..

[12] Okawa S., Chirwa M., Ishikawa N., Kapyata H., Msiska C.Y., Syakantu G., Miyano S., Komada K., Jimba M., Yasuoka J. (2015). Longitudinal adherence to antiretroviral drugs for preventing mother-to-child transmission of HIV in Zambia. BMC Pregnancy Childbirth.

[13] Llenas-García J., Wikman-Jorgensen P., Hobbins M., Mussa M.A., Ehmer J., Keiser O., Mbofana F., Wandeler G., SolidarMed and IeDEA-Southern Africa (2016). Retention in care of HIV-infected pregnant and lactating women starting ART under Option B+ in rural Mozambique. Trop. Med. Int. Health.

[14] Dzangare J., Takarinda K.C., Harries A.D., Tayler-Smith K., Mhangara M., Apollo T.M., Mushavi A., Chimwaza A., Sithole N., Magure T. (2016). HIV testing uptake and retention in care of HIV-infected pregnant and breastfeeding women initiated on ‘Option B+’ in rural Zimbabwe. Trop. Med. Int. Health.

[15] Erlwanger A.S., Joseph J., Gotora T., Muzunze B., Orne-Gliemann J., Mukungunugwa S., Farley T., Mangwiro A.-Z. (2017). Patterns of HIV Care Clinic Attendance and Adherence to Antiretroviral Therapy Among Pregnant and Breastfeeding Women Living With HIV in the Context of Option B+ in Zimbabwe. J. Acquir. Immune Defic. Syndr..

[16] Rollins N.C., Essajee S.M., Bellare N., Doherty M., Hirnschall G.O. (2017). Improving Retention in Care Among Pregnant Women and Mothers Living With HIV: Lessons from INSPIRE and Implications for Future WHO Guidance and Monitoring. J. Acquir. Immune Defic. Syndr..

[17] Clouse K., Schwartz S., van Rie A., Bassett J., Yende N., Pettifor A. (2014). What they wanted was to give birth; nothing else: Barriers to retention in option B+ HIV care among postpartum women in South Africa. J. Acquir. Immune Defic. Syndr..

[18] Tweya H., Gugsa S., Hosseinipour M., Speight C., Ng’Ambi W., Bokosi M., Chikonda J., Chauma A., Khomani P., Phoso M. (2014). Understanding factors, outcomes and reasons for loss to follow-up among women in Option B+ PMTCT programme in Lilongwe, Malawi. Trop. Med. Int. Health.

[19] Koo K., Makin J.D., Forsyth B.W. (2013). Barriers to male-partner participation in programs to prevent mother-to-child HIV transmission in South Africa. AIDS Educ. Prev..

[20] Colvin C.J., Konopka S., Chalker J.C., Jonas E., Albertini J., Amzel A., Fogg K. (2014). A systematic review of health system barriers and enablers for antiretroviral therapy (ART) for HIV-infected pregnant and postpartum women. PLoS ONE.

[21] Zacharius K.M., Basinda N., Marwa K., Mtui E.H., Kalolo A., Kapesa A. (2019). Low adherence to Option B+ antiretroviral therapy among pregnant women and lactating mothers in eastern Tanzania. PLoS ONE.

[22] Adeniyi V., Ajayi A.I., Goon D.T., Owolabi E.O., Eboh A., Lambert J. (2018). Factors affecting adherence to antiretroviral therapy among pregnant women in the Eastern Cape, South Africa. BMC Infect. Dis..

[23] Kim M.H., Zhou A., Mazenga A., Ahmed S., Markham C., Zomba G., Simon K., Kazembe P.N., Abrams E.J. (2016). Why did I stop? Barriers and facilitators to uptake and adherence to ART in Option B+ HIV care in Lilongwe, Malawi. PLoS ONE.

[24] Ministry of Health (Malawi Government) (2015). Malawi ARV Patient Education Flip Chart. https://www.tingathe.org/uploads/8/8/3/0/88301718/malawi_arv_patient_education_flipchart_general.pdf.

[25] Kim M.H., Tembo T.A., Mazenga A., Yu X., Myer L., Sabelli R., Flick R., Hartig M., Wetzel E., Simon K. (2020). The Video intervention to Inspire Treatment Adherence for Life (VITAL Start): Protocol for a multisite randomized controlled trial of a brief video-based intervention to improve antiretroviral adherence and retention among HIV-infected pregnant women in Malawi. Trials.

[26] Kim M.H., Ahmed S., Tembo T., Sabelli R., Flick R., Yu X., Mazenga A., le Blond H., Simon K., Hartig M. (2019). VITAL Start: Video-Based intervention to Inspire Treatment Adherence for Life—Pilot of a novel video-based approach to HIV counseling for pregnant women living with HIV. AIDS Behav..

[27] Kjellsson G., Clarke P., Gerdtham U.-G. (2014). Forgetting to remember or remembering to forget: A study of the recall period length in health care survey questions. J. Health Econ..

[28] Brown M., Wexler C., Gautney B., Goggin K., Hurley E.A., Odeny B., Maloba M., Lwembe R., Sandbulte M., Finocchario-Kessler S. (2019). eHealth Interventions for Early Infant Diagnosis: Mothers’ Satisfaction with the HIV Infant Tracking System in Kenya. AIDS Behav..

[29] Powell R.E., Henstenburg J.M., Cooper G., Hollander J.E., Rising K.L. (2017). Patient Perceptions of Telehealth Primary Care Video Visits. Ann. Fam. Med..

[30] Ulin P.R., Robinson E.T., Tolley E.E. (2005). Qualitative Methods in Public Health.

[31] Patton M.Q. (2015). Qualitative Research & Evaluation Methods: Integrating Theory and Practice.

[32] Rothstein J.D., Jennings L., Moorthy A., Yang F., Gee L., Romano K., Hutchful D., Labrique A.B., Lefevre A.E. (2016). Qualitative Assessment of the Feasibility, Usability, and Acceptability of a Mobile Client Data App for Community-Based Maternal, Neonatal, and Child Care in Rural Ghana. Int. J. Telemed. Appl..

[33] Wanga I., Helova A., Abuogi L.L., Bukusi E.A., Nalwa W., Akama E., Odeny T.A., Turan J.M., Onono M. (2019). Acceptability of community-based mentor mothers to support HIV-positive pregnant women on antiretroviral treatment in western Kenya: A qualitative study. BMC Pregnancy Childbirth.

[34] Proctor E., Silmere H., Raghavan R., Hovmand P., Aarons G., Bunger A., Griffey R., Hensley M. (2011). Outcomes for implementation research: Conceptual distinctions, measurement challenges, and research agenda. Adm. Policy Ment. Health.

[35] McCarthy J., Blanc A.K., Warren C.E., Mdawida B. (2018). Women’s recall of maternal and newborn interventions received in the postnatal period: A validity study in Kenya and Swaziland. J. Glob. Health.

[36] Krakowiak P., Walker C.K., Tancredi D.J., Hertz-Picciotto I. (2015). Maternal recall versus medical records of metabolic conditions from the prenatal period: A validation study. Matern. Child Health J..

[37] Moskowitz J.T., Carrico A.W., Duncan L.G., Cohn M.A., Cheung E.O., Batchelder A., Martinez L., Segawa E., Acree M., Folkman S. (2017). Randomized controlled trial of a positive affect intervention for people newly diagnosed with HIV. J. Consult. Clin. Psychol..

[38] Bland R.M., Rollins N.C., Solarsh G., van den Broeck J., Coovadia H.M. (2003). Maternal recall of exclusive breast feeding duration. Arch. Dis. Child..

[39] Bahrick H.P. (1979). Maintenance of knowledge: Questions about memory we forgot to ask. J. Exp. Psychol. Gen..

[40] Tedeschi R.G., Calhoun L.G. (2004). Posttraumatic growth: Conceptual foundations and empirical evidence. Psychol. Inq..

[41] Nightingale V.R., Sher T.G., Hansen N.B. (2010). The impact of receiving an HIV diagnosis and cognitive processing on psychological distress and posttraumatic growth. J. Trauma. Stress.

[42] Hekkenberg R.J., Irish J.C., Rotstein L.E., Brown D.H., Gullane P.J. (1997). Informed consent in head and neck surgery: How much do patients actually remember?. J. Otolaryngol..

[43] Kennedy C.E., Fonner V.A., Armstrong K.A., O’reilly K.R., Sweat M.D. (2015). Increasing HIV serostatus disclosure in low-and middle-income countries: A systematic review of intervention evaluations. AIDS (Lond. UK).

[44] Mekonnen A., Lakew A.M., Muchie K.F., Teshome D.F. (2019). Sero-positive HIV result disclosure to sexual partner in Ethiopia: A systematic review and meta-analysis. BMC Public Health.

[45] Broeckaert L., Challacombe L. (2016). Disclosure Programming: A Review of the Evidence. Prev. Focus.

[46] Futterman D.C., Shea J., Besser M., Stafford S., Desmond K., Comulada W.S., Greco E. (2010). Mamekhaya: A pilot study combining a cognitive-behavioral intervention and mentor mothers with PMTCT services in South Africa. AIDS Care.

[47] Jones D., Peltzer K., Villar-Loubet O., Shikwane E., Cook R., Vamos S., Weiss S.M. (2013). Reducing the risk of HIV infection during pregnancy among South African women: A randomized controlled trial. AIDS Care.

[48] Sarnquist C.C., Moyo P., Stranix-Chibanda L., Chipato T., Kang J.L., Maldonado Y.A. (2014). Integrating family planning and prevention of mother to child HIV transmission in Zimbabwe. Contraception.

[49] Evangeli M., Wroe A.L. (2017). HIV disclosure anxiety: A systematic review and theoretical synthesis. AIDS Behav..

[50] Tam M., Amzel A., Phelps B.R. (2015). Disclosure of HIV serostatus among pregnant and postpartum women in sub-Saharan Africa: A systematic review. AIDS Care.

[51] Adeoye-Agboola D., Evans H., Hewson D., Pappas Y. (2016). Factors influencing HIV disclosure among people living with HIV/AIDS in Nigeria: A systematic review using narrative synthesis and meta-analysis. Public Health.

[52] WHO Gender Dimensions of HIV Status Disclosure to Sexual Partners: Rates, Barriers and Outcomes; 2004; Volume 2020. https://apps.who.int/iris/handle/10665/42717.

[53] Artz L., Macaluso M., Kelaghan J., Austin H., Fleenor M., Robey L., Hook E.W., Brill I. (2005). An intervention to promote the female condom to sexually transmitted disease clinic patients. Behav. Modif..

[54] Besera G.T., Cox S., Malotte C.K., Rietmeijer C.A., Klausner J.D., O’Donnell L., Margolis A.D., Warner L. (2016). Assessing Patient Exposure to a Video-Based Intervention in STD Clinic Waiting Rooms: Findings From the Safe in the City Trial. Health Promot. Pract..

[55] Myint-U A., Bull S., Greenwood G.L., Patterson J., Rietmeijer C.A., Vrungos S., Warner L., Moss J., O’Donnell L.N. (2010). Safe in the city: Developing an effective video-based intervention for STD clinic waiting rooms. Health Promot. Pract..

[56] Warner L., Klausner J.D., Rietmeijer C.A., Malotte C.K., O’Donnell L., Margolis A.D., Greenwood G.L., Richardson D., Vrungos S., O’Donnell C.R. (2008). Effect of a Brief Video Intervention on Incident Infection among Patients Attending Sexually Transmitted Disease Clinics. PLoS Med..

[57] Bonsignore L., Bloom N., Steinhauser K., Nichols R., Allen T., Twaddle M., Bull J. (2018). Evaluating the Feasibility and Acceptability of a Telehealth Program in a Rural Palliative Care Population: TapCloud for Palliative Care. J. Pain Symptom Manag..

[58] Coetzee B.J., Ms H.K., Tomlinson M., Ma N.M., Le Roux I., Adam M. (2018). Community health workers’ experiences of using video teaching tools during home visits-A pilot study. Health Soc. Care Community.

[59] Calderon Y., Leider J., Hailpern S., Haughey M., Ghosh R., Lombardi P., Bijur P., Bauman L. (2009). A randomized control trial evaluating the educational effectiveness of a rapid HIV posttest counseling video. Sex. Transm. Dis..

[60] Staley S.A., Charm S.S., Slough L.B., Zerden M.L., Morse J.E. (2019). Prenatal Contraceptive Counseling by Video. South. Med. J..

[61] Cox J., Gutner C., Kronfli N., Lawson A., Robbins M., Nientker L., Ostawal A., Barber T.J., Croce D., Hardy D. (2019). A need for implementation science to optimise the use of evidence-based interventions in HIV care: A systematic literature review. PLoS ONE.

[62] Queirós A., Faria D., Almeida F. (2017). Strengths and limitations of qualitative and quantitative research methods. Eur. J. Educ. Stud..

[63] Taylor S.J., Bogdan R., DeVault M. (2015). Introduction to Qualitative Research Methods: A Guidebook and Resource.

[64] National Statistical Office (NSO) [Malawi] and ICF (2017). Malawi Demographic and Health Survey 2015-16.

